# The Burden of Gastrointestinal Anastomotic Leaks: an Evaluation of Clinical and Economic Outcomes

**DOI:** 10.1007/s11605-014-2506-4

**Published:** 2014-03-27

**Authors:** Jeffrey Hammond, Sangtaeck Lim, Yin Wan, Xin Gao, Anuprita Patkar

**Affiliations:** 1 Medical Affairs, Ethicon, Inc, Somerville, NJ 08876 USA; 2 Global Health Economics and Market Access, Ethicon, Inc, Somerville, NJ 08876 USA; 3 Health Outcomes Research, Pharmerit North America LLC, Bethesda, MD 20814 USA

**Keywords:** Postoperative anastomotic leak, Colorectal surgery, Health economics, Length of stay, Cost

## Abstract

**Objective:**

To evaluate the clinical and economic burden associated with anastomotic leaks following colorectal surgery.

**Methods:**

Retrospective data (January 2008 to December 2010) were analyzed from patients who had colorectal surgery with and without postoperative leaks, using the Premier Perspective™ database. Data on in-hospital mortality, length of stay (LOS), re-admissions, postoperative infection, and costs were analyzed using univariate and multivariate analyses, and the propensity score matching (PSM) and generalized linear models (GLM).

**Results:**

Of the patients, 6,174 (6.18 %) had anastomotic leaks within 30 days after colorectal surgery. Patients with leaks had 1.3 times higher 30-day re-admission rates and 0.8–1.9 times higher postoperative infection rates as compared with patients without leaks (*P* < 0.001 for both). Anastomotic leaks incurred additional LOS and hospital costs of 7.3 days and $24,129, respectively, only within the first hospitalization. Per 1,000 patients undergoing colorectal surgery, the economic burden associated with anastomotic leaks—including hospitalization and re-admission—was established as 9,500 days in prolonged LOS and $28.6 million in additional costs. Similar results were obtained from both the PSM and GLM for assessing total costs for hospitalization and re-admission.

**Conclusions:**

Anastomotic leaks in colorectal surgery increase the total clinical and economic burden by a factor of 0.6–1.9 for a 30-day re-admission, postoperative infection, LOS, and hospital costs.

## Introduction

Anastomotic leaks are one of the most serious complications that occur after gastrointestinal surgery. They add to potential postoperative patient morbidities and to overall costs of postoperative patient care, including associated hospital re-admissions. Further, reoperations and complications such as leaks are considered a quality indicator in colorectal surgery.^1^

Patients developing anastomotic leaks after undergoing colorectal resection exhibit poorer long-term functional results; in the case of malignancy, increased local recurrence rates and reduced 5-year survival are seen.^2^–^4^ The clinical manifestations of anastomotic leaks will often warrant hospital re-admission, placing a considerable additional burden on patients and healthcare providers. Overall, anastomotic leaks after colorectal surgery have devastating implications, with significantly greater chances of wound infection and mortality rates of up to 32 %.^5^, ^6^

In addition to potential negative clinical outcomes, there is a significant economic and healthcare utilization burden to be considered. While postoperative complications have a dramatic impact on full in-hospital costs per case and are the strongest indicator of costs,^7^ there remains a gap in the literature in pairing clinical sequelae of postoperative anastomotic leaks to economic outcomes.

Reported leak rates for colorectal surgery range from 1.5 to 16 % globally; however, definitions of leaks differ between published studies.^8^ Furthermore, a review by Kingham and Pachter reported that experienced colorectal surgeons often quote 3 to 6 % as a generally acknowledged overall leakage rate. They also compared the definitions across different studies and concluded that there was no uniformly accepted set of criteria.^9^ They observed that definitions varied based on combinations of clinical signs, biochemical markers, radiological findings, and intraoperative findings. Our focus was on clinical leaks, as they affect morbidity and mortality. Nonclinical leaks diagnosed by radiology have no clinical effects and resolve without interventions. Our study was undertaken to quantify the incidence of anastomotic leaks in patients undergoing colorectal surgery and to assess the clinical and economic burden of anastomotic leaks in terms of extended hospital stay, re-admissions, in-hospital mortality, postoperative infection, and total costs following colorectal surgery.

## Methods

### Study Design

This study was designed as a retrospective data analysis of hospital-based patients to analyze the health outcomes and medical resource utilization of patients with anastomotic leaks following colorectal surgery.

### Database and Sample Selection

Matched and unmatched cohort studies were conducted retrospectively, utilizing hospital administrative data from the Premier Perspective™ database (Premier, Inc., Charlotte, NC, USA) from January 2008 to December 2010.^10^ This database contains complete clinical coding, hospital cost, and patient billing data from more than 600 hospitals throughout the USA. Furthermore, it collects data from participating hospitals in its healthcare alliance. The Premier healthcare alliance was formed for hospitals to share knowledge, improve patient safety, and reduce risks. Participation in the Premier healthcare alliance is voluntary. The hospitals included are nationally representative based on bed size, geographic location, and teaching hospital status.^11^ The database contains a date-stamped log of all billed items by the cost-accounting department, including medications; laboratory, diagnostic, and therapeutic services; and primary and secondary diagnoses for each patient’s hospitalization. Identifier-linked enrollment files provide demographic and payer information. Detailed service-level information for each hospital day was recorded; this included details on medication received.

The patient information collected included patient demographics (age, gender, race/ethnicity), clinical characteristics (All Patient Refined Diagnosis Related Groups [APR-DRG] severity, APR-DRG risk of mortality, Charlson Comorbidity Index [CCI]), hospital and admitting characteristics (location and region of hospital, number of beds, teaching status, admission type), primary and secondary diagnoses, primary and secondary procedures, payer, length of stay (LOS), cost of care, drug utilization, department cost and charge details, day-of-stay capture for some variables, and physician specialty. The APR-DRG, a widely accepted healthcare research methodology developed by 3M, is an indicator of the severity degree of a disease; it is classified into four categories—minor, moderate, major, and extreme. For the purpose of this study, Version 26 of the APR-DRG was used. The CCI is a tool used to predict the 10-year mortality for a patient who may have a range of comorbid conditions. Data from all payer types were included. Laboratory/culture data were not available for this study.

### Inclusion/Exclusion Criteria

Data on patients ≥18 years of age, who had undergone surgery on the colon or rectum and with colon or rectal anastomosis, were included in the study. All eligible patients underwent colorectal surgery, first performed during the study period. The surgeries considered for inclusion were colectomy, hemicolectomy, or rectum resection, and were identified by International Classification of Diseases, version 9 (ICD-9) procedure codes, Current Procedural Terminology (CPT) codes, and Perspective Standard Charge Master codes. Excluded from the study were patients who were under 18 years of age, admitted for trauma-related diagnoses, had an LOS of 0 during the timeframe under study, and patients with a missing index colorectal surgery day. Also excluded were patients with a protective ostomy, i.e., an ostomy as part of the index procedure.

### Outcome Measures

The primary objectives of this study were to examine the incidence of anastomotic leaks following colorectal surgery and to evaluate the associated clinical and economic burden. Anastomotic leaks were defined by re-operation, re-anastomosis, stent, colostomy, drainage, and/or abscess within a 30-day window following colorectal surgery, and were identified by ICD-9 procedure codes, CPT codes, and Perspective Standard Charge Master codes. These re-intervention codes were selected because they are specific or suggestive of procedures surgeons would follow when faced with a clinically apparent anastomotic leak.

The total costs of hospitalization, including re-admission (if applicable), and by-department costs, were recorded and analyzed. The cost variable we chose represented the actual cost to treat the patient and included all supplies, labor, and depreciation of equipment. The cost was calculated by adding the variable costs (direct) and the fixed costs (overhead) in the Premier Perspective™ database. In addition, data were analyzed on in-hospital mortality (hospitalization and re-admission, individually and combined) and postoperative infection during hospitalization. The definitions used for postoperative infection were based on ICD-9 codes 998.5X and 998.6X, and on nonprophylactic antibiotic usage as a proxy for postoperative infections, defined as postoperative exposure to the predefined antimicrobial drugs ≥2 days after surgery and with treatment duration ≥7 days.^12^ Data on discharge status were recorded, including whether patients were discharged to their home, a skilled nursing facility, other institutions, or to a short-term general hospital; unknown reasons and patients’ death were also recorded within the discharge data. Data concerning hospital LOS and re-admission within 30 days after discharge were recorded individually and combined.

Covariates used for the analysis included anastomotic leaks (as defined previously); age; race; gender; admission type; CCI; type of colorectal index surgery (left colon/sigmoid, right colon, colorectal); APR-DRG severity level; APR-DRG risk of mortality; and hospital region, location, type, and size.

### Statistical Analysis

#### Univariate Analysis

Descriptive analyses were conducted to show the overall incidence of anastomotic leaks among the cohorts, patient baseline demographic and clinical characteristics, and hospital characteristics. Univariate analyses were performed to assess the unadjusted difference in outcomes and covariates between the two groups. Continuous measures (e.g., age) were summarized by mean and standard deviation, and comparisons of the differences in continuous measures between study groups were made using the student *t* tests. Categorical variables were summarized as proportions of the sample with the characteristic and compared using chi-squared tests between study groups. Comparison of continuous variables without normal distribution (e.g., cost data) was performed using a Wilcoxon–Mann–Whitney nonparametric test.

#### Propensity Score Matching

The differences in the clinical and economic outcomes were compared by adjusting for covariates using the propensity score matching (PSM) method. The PSM method matched a patient without anastomotic leaks to each of the patients with leaks, using a 1:1 ratio and the nearest neighbor matching algorithm, Parsons, which is the most frequently used case/control matching algorithm.^13^ In addition, the nearest available pair matching method was used. The cases were ordered and sequentially matched to the nearest unmatched control. If more than one unmatched control matched a case, the control was selected at random. PSM utilizes the baseline characteristics into a single index variable to facilitate the desired matching. The likelihood of having an anastomotic leak was estimated based on individual characteristics for each patient with and without anastomotic leaks, using a binary logistic regression model. Each case of anastomotic leaks was then matched with a patient from the nonleak cohort who had the closest propensity score to that of the case’s score. After matching, comparisons between the cohorts were performed on covariates and outcome variables using various statistical tests as described above.

#### Multivariate Analysis

Generalized linear models (GLMs) were used to describe the impact of the anastomotic leaks on economic outcomes in patients with colorectal surgery, thereby confirming the results from the PSM method on the economic outcomes. GLMs are empirical transformations of classical linear (Gaussian) regression models and are distinguished from Ordinary Least Squares (OLS) by a particular model, rather than data transformations. Healthcare expenditure and resource use data frequently have a log-normal or gamma distribution, and studies using GLM for cost and resource use analysis have focused on the gamma response distribution.^14^ GLMs are the preferred approach for multivariate analysis of cost data. In order to analyze LOS data, negative binomial distribution was used. All analyses were conducted using SAS version 9.2 (SAS Institute Inc., Cary, NC, USA). *P* values ≤0.05 were considered statistically significant.

## Results

### Baseline Characteristics

**Fig. 1:**
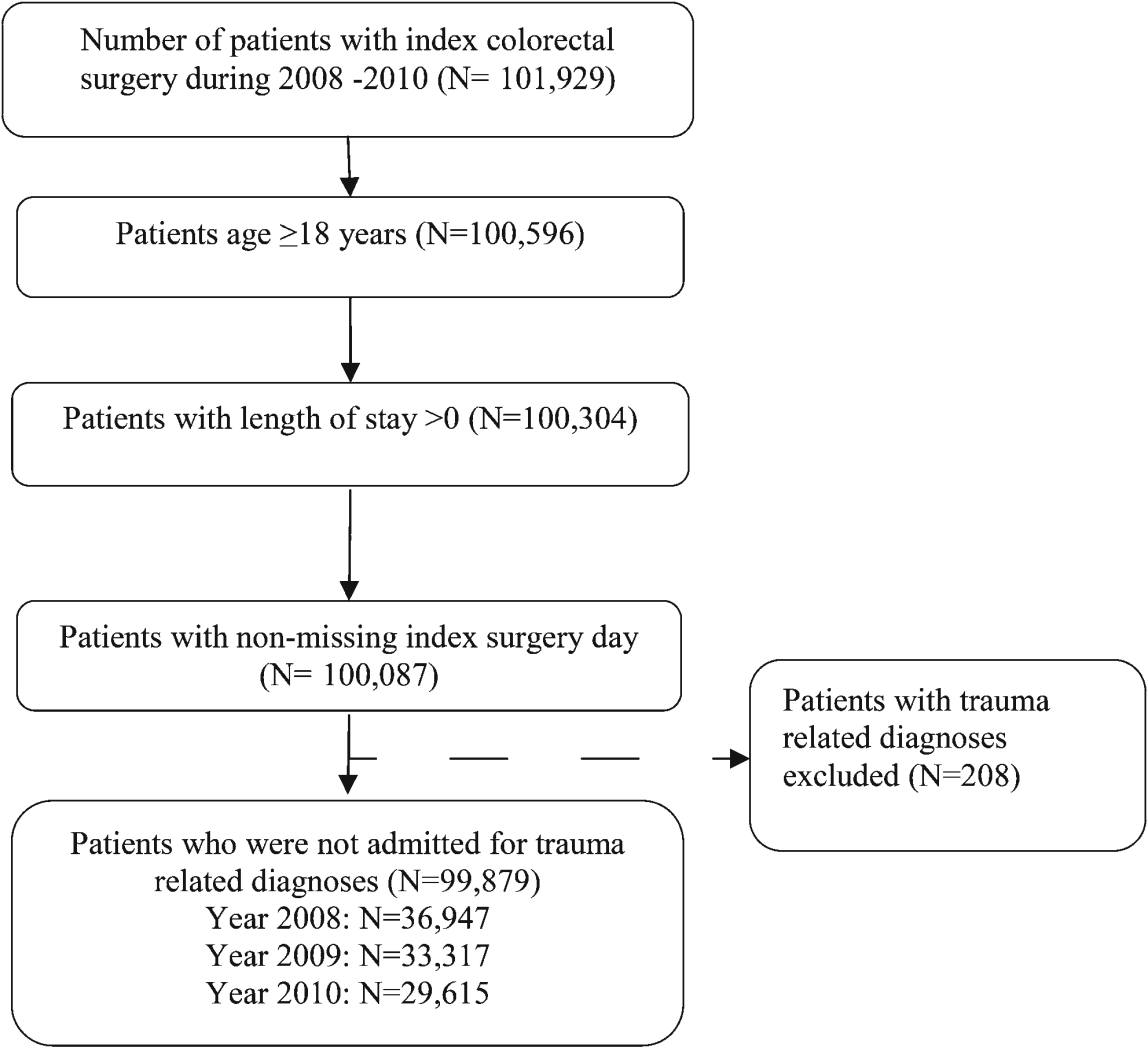
Patient selection

Records of 101,929 patients who underwent colorectal surgery from 2008 to 2010 were screened. A total of 99,879 records fit the inclusion criteria (Fig. 1). The mean (SD) age of the patients was 63.1 (15.3) years, with 54 % female and 46 % male. Differences were noted in baseline demographic and clinical characteristics (Tables 1 and 2), and in age, sex, race, and health plans between patients with and without anastomotic leaks (*P* < 0.001 for all). Additionally, significant differences in clinical characteristics were observed between patients with and without leaks, including APR-DRG severity, CCI, individual comorbidities, and surgery type. The overall incidence rate for 30-day postoperative anastomotic leaks was 6.18 % (6,174 patients). Annual mean leak rates for 2008, 2009, and 2010 were 5.69, 6.46, and 6.48 %, respectively. The mean leak rate was higher for surgeries around the colorectal area (6.54 %) than for those on the left (5.82 %) or right (6.09 %) colon alone.

**Table 1:** Demographics of patients with and without anastomotic leak

Variable		Overall (*N* = 99,879)	Patients without anastomotic leak (*N* = 93,705)	Patients with anastomotic leak (*N* = 6,174)	*P* value
Age, mean (SD)		63.1 (15.3)	63.2 (15.3)	61.2 (15.8)	<0.001
Age					
18–44	No. (%)	11,831 (12)	10,875 (12)	956 (15)	<0.001
45–54		16,703 (17)	15,693 (17)	1,010 (16)	
55–64		22,240 (22)	20,847 (22)	1,393 (23)	
65+		49,105 (49)	46,290 (49)	2,815 (46)	
Sex					
Female	No. (%)	54,080 (54)	51,052 (54)	3,028 (49)	<0.001
Male		45,796 (46)	42,650 (46)	3,146 (51)	
Race					
White	No. (%)	69,610 (70)	65,406 (70)	4,204 (68)	<0.001
Black		9,383 (9)	8,670 (9)	713 (12)	
Other		20,886 (21)	19,629 (49)	2,815 (46)

**Table 2:** Baseline clinical characteristics of patients with and without anastomotic leak

Variable		Overall (*N* = 99,879)	Patients without anastomotic leak (*N* = 93,705)	Patients with anastomotic leak (*N* = 6,174)	*P* value
Severity level of a patient for a specific APR-DRG					
Minor	No. (%)	23,539 (24)	23,162 (25)	377 (6)	<0.001
Moderate		34,029 (34)	33,055 (35)	974 (16)	
Major		24,386 (24)	22,801 (24)	1,585 (26)	
Extreme		17,925 (18)	14,687 (16)	3,238 (52)	
APR-DRG risk of mortality					
Minor	No. (%)	42,464 (43)	41,412 (44)	1,052 (17)	<0.001
Moderate		25,583 (26)	24,506 (26)	1,077 (17)	
Major		17,433 (17)	15,967 (17)	1,466 (24)	
Extreme		14,399 (14)	11,820 (13)	2,579 (42)	
Charlson Comorbidity Index, mean (SD)		2.5 (3.0)	2.5 (3.0)	2.6 (3.1)	<0.001
Comorbidity					
Comorbidity 0	No. (%)	35,457 (35)	33,423 (36)	2,034 (33)	<0.001
Comorbidity ≥1		64,422 (65)	60,282 (64)	4,140 (67)	
Type of index surgery					
Left colon/sigmoid	No. (%)	37,798 (38)	35,598 (38)	2,200 (36)	<0.001
Right colon		19,596 (20)	18,402 (20)	1,194 (19)	
Colorectal		42,485 (43)	39,705 (42)	2,780 (45)	
Admission type					
Emergency	No. (%)	31,198 (31)	28,448 (30)	2,750 (45)	<0.001
Urgent		10,370 (10)	9,568 (10)	802 (13)	
Elective		57,969 (58)	55,375 (59)	2,594 (42)	
Other		342 (0)	314 (0)	28 (0)	
Health plan					
Medicare	No. (%)	49,119 (49)	46,206 (49)	2,913 (47)	<0.001
Medicaid		5,218 (5)	4,704 (5)	514 (8)	
Commercial		38,988 (39)	36,793 (39)	2,195 (36)	
Other		6,554 (7)	6,002 (6)	552 (9)

**Table 3:** Clinical outcomes of patients with anastomotic leakage and matched control patients

Variable		Overall (*N* = 12,348)	Patients without anastomotic leak (*N* = 6,174)	Patients with anastomotic leak (*N* = 6,174)	*P* value
In-hospital mortality for index hospitalization ONLY, No. (%)		1,295 (10)	649 (11)	646 (10)	0.93
Mortality of re-admission ONLY, No. (%)		114 (4)	46 (6)	68 (4)	0.014
Mortality of index hospitalization and re-admission, No. (%)		1,409 (11)	695 (11)	714 (12)	0.59
Discharge					
Home	No. (%)	4,397 (36)	2,500 (40)	1,897 (31)	<0.001
Home with nursing care		3,120 (25)	1,425 (23)	1,695 (27)	
Nursing facility		3,221 (26)	1,481 (24)	1,740 (28)	
Expired		1,295 (10)	649 (11)	646 (10)	
Other facility		309 (3)	116 (2)	193 (3)	
Unknown		6 (0)	3 (0)	3 (0)	
Postoperative infection, No. (%)		2,271 (18)	586 (9)	1,685 (27)	<0.001
Infection defined by nonprophylactic antibiotic usage, No. (%)		25,079 (25)	20,949 (22)	4,130 (67)	<0.001

### Clinical Outcomes

#### Mortality and Discharge Status

Univariate analysis of clinical outcomes showed significant differences in in-hospital mortality for patients undergoing index hospitalization or re-admission; when combined, mortality was reported in 12 % of patients with anastomotic leaks as compared to 4 % of patients without leaks (*P* < 0.001). However, after PSM, there were no statistically significant differences in mortality between the two cohorts. The PSM analysis showed significant differences in the discharge status—the proportions of patients with and without anastomotic leaks with a discharge status of “home,” “home with nursing care,” and “nursing facility” were 31 and 40 %, 27 and 23 %, and 28 and 24 %, respectively (Table 3).

#### Postoperative Infection

**Fig. 2:**
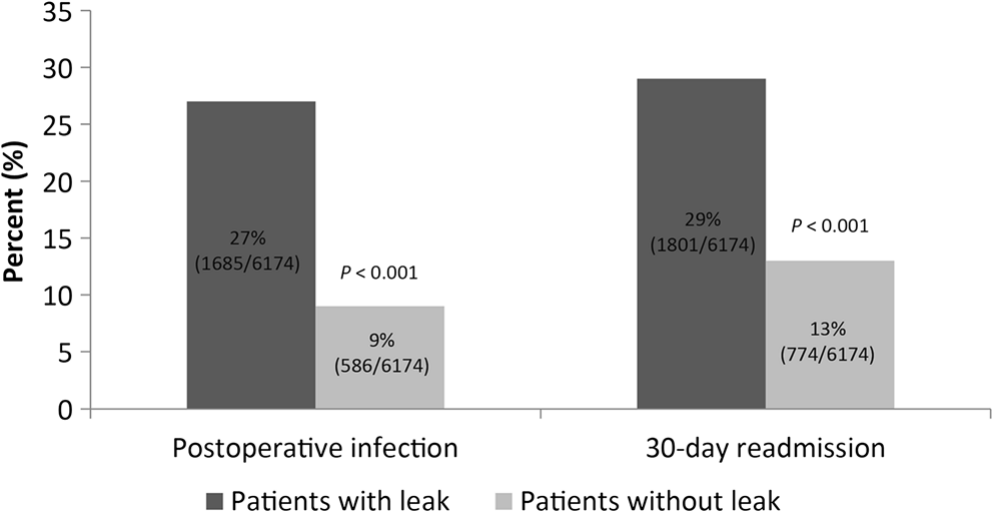
Postoperative infection and subsequent 30-day re-admission reported in two groups of patients

Postoperative infection was reported in 27 and 9 % of patients with and without leaks, respectively (Fig. 2). Patients with leaks reported a postoperative infection rate that was 0.8–1.9 times higher than that of patients without leaks (*P* < 0.001). The subsequent 30-day re-admission was reported in 29 % and 13 % of patients with and without leaks, respectively (*P* < 0.001; Fig. 2). When defined by nonprophylactic antibiotic use only, 58 and 33 % of patients with and without leaks, respectively, reported postoperative infection (*P* < 0.001). Table 3 examines the clinical outcomes of patients with leaks and matched controls.

#### Reasons for Re-admission

The primary reasons for re-admission of patients with and without anastomotic leaks included surgical site infection (25 vs 10 %), and gastrointestinal (22 % in both groups) and genitourinary (5 vs 7 %) causes. Overall, 14 % of re-admissions were related to complications of surgical and medical care. Table 4 shows the primary reasons for re-admission in patients with and without leaks.

**Table 4:** Reasons for a 30-day re-admission for patients with and without anastomotic leak

Primary diagnoses	Total (*N* = 11,079)	Patients without anastomotic leak (*N* = 9,278)	Patients with anastomotic leak (*N* = 1,801)
Gastrointestinal complications, No. (%)	2,393 (22)	2,001 (22)	392 (22)
Surgical site infection, No. (%)	1,380 (12)	935 (10)	445 (25)
Genitourinary, No. (%)	767 (7)	671 (7)	96 (5)
Cardiac/circulatory, No. (%)	524 (5)	486 (5)	38 (2)
Venous thromboembolism, No. (%)	819 (7)	733 (8)	86 (5)
Other infections, No. (%)^a^	769 (7)	661 (7)	108 (6)
Neurologic/nervous system, No. (%)	210 (2)	192 (2)	18 (1)
Aftercare and services for specific procedures, No. (%)	1,310 (12)	1,194 (13)	116 (6)
Complication of surgical and medical care, not elsewhere classified, No. (%)	1,528 (14)	1,172 (13)	356 (20)
Others, No. (%)^b^	2,146 (19)	1,904 (21)	242 (13)

^a^Other infections include septicemia- and intestinal infection-related complications

^b^Others include neoplasm-, endocrine-, and genitourinary-related complications

### Economic Outcomes and Hospitalization

The unmatched univariate analysis showed significant differences in mean LOS between patients with and without leaks (23 vs 9.7 days; *P* < 0.001). For all patients with and without leaks, the mean (SD) inpatient cost, including index hospitalization and re-admission, was $27,966 ($38,609), with a significant difference between mean costs observed in patients with and without anastomotic leaks ($72,905 [$94,723] vs $25,005 [$29,256], respectively; *P* < 0.01).

**Fig. 3:**
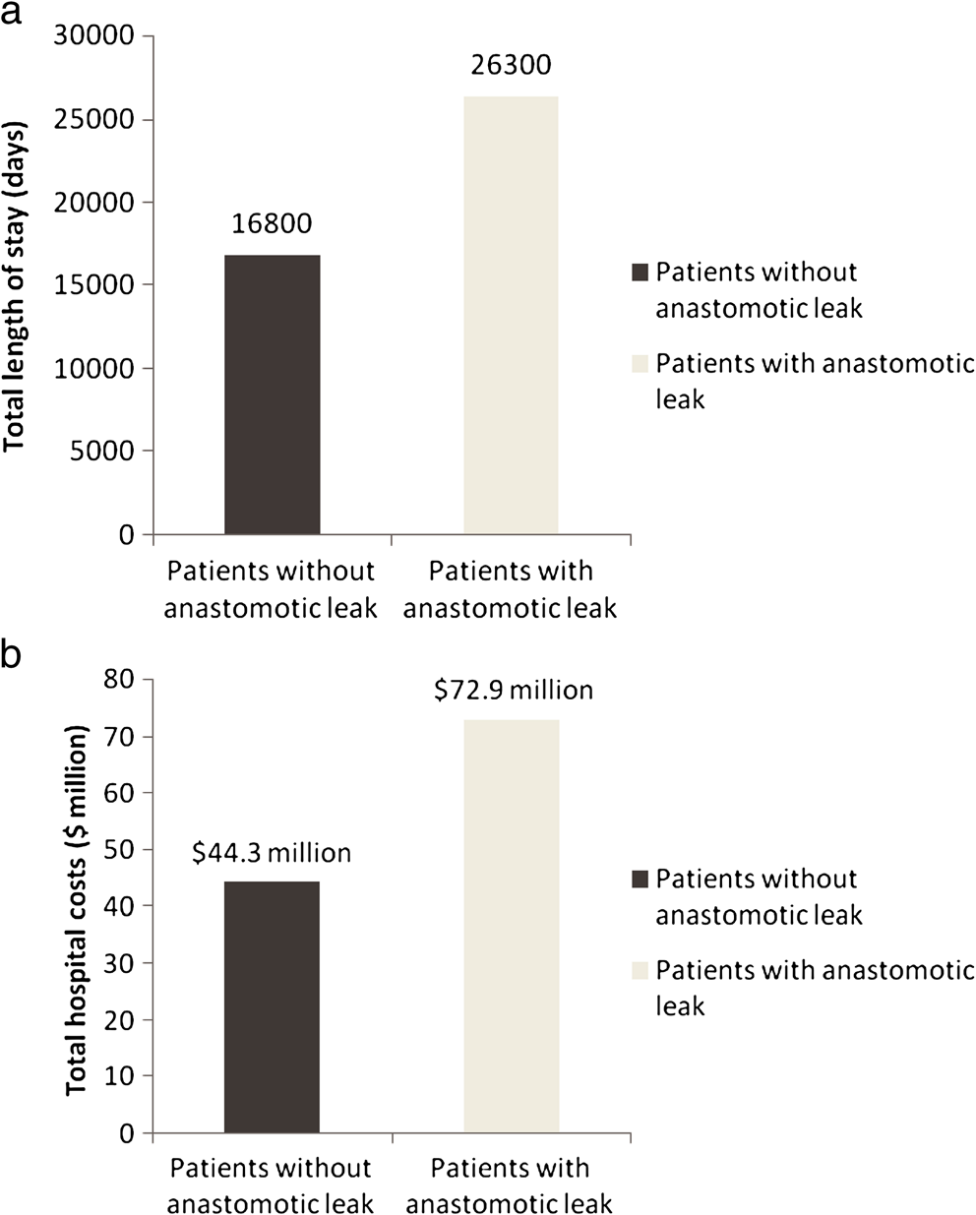
**a** Total length of stay and **b** total hospital cost per 1,000 patients with and without anastomotic leak

After PSM, a number of significant differences were observed in economic outcomes between patients with and without leaks (Table 5). For patients with leaks, additional average LOS increases of 7.3 days and hospital costs of $24,129 were incurred for hospitalization alone. Patients with leaks had a 1.3-fold greater chance of a 30-day re-admission. After factoring in re-admissions, the average incremental LOS and average incremental hospital cost increased by up to 9.5 days and $28,597, respectively. The total LOS per 1,000 patients was 26,300 and 16,800 days in patients with and without leaks, respectively (Fig. 3a). The resulting total hospital costs per 1,000 patients were $72.9 million and $44.3million for patients with and without leaks, respectively (Fig. 3b). Population-level results demonstrated that incremental burdens of anastomotic leaks in hospitalization and re-admissions resulted in 9,500 days of increased LOS and $28.6 million in additional payments.

**Table 5:** Economic outcomes of patients with anastomotic leakage and matched control patients

Variable	Overall (*N* = 12,348)	Patients without anastomotic leak (*N* = 6,174)	Patients with anastomotic leak (*N* = 6,174)	*P* value
30-day re-hospitalization, No. (%)	2,575 (21)	774 (13)	1,801 (29)	<0.001
Length of stay for index hospitalization ONLY, mean (SD)	19.3 (20.6)	15.7 (16.7)	23.0 (23.3)	<0.001
Total length of stay, re-admission ONLY, mean (SD)	10.6 (12.2)	8.8 (9.6)	11.3 (13.1)	<0.001
Total length of stay, including index and re-admission BOTH, mean (SD)	21.5 (22.0)	16.8 (17.9)	26.3 (24.6)	<0.001
Total cost ($) for index hospitalization ONLY, mean (SD)	54,415 (76,043)	42,350 (50,172)	66,479 (93,583)	<0.001
Total inpatient cost ($), re-admission ONLY, mean (SD)	20,101 (25,568)	15,619 (21,206)	22,028 (27,006)	<0.001
Total inpatient cost ($), including index and re-admission BOTH, mean (SD)	58,607 (77,788)	44,308 (52,168)	72,905 (94,723)	<0.001
Cost ($) by department-lab, mean (SD)	4,150.6 (6,288.9)	3,225.2 (5,123.2)	5,076.0 (7,151.7)	<0.001
Cost ($) by department-pharmacy, mean (SD)	8,320.7 (41,581)	5,849.4 (9276.5)	10,792 (57,965)	<0.001
Cost ($) by department-radiology, mean (SD)	1,825.3 (2,424.4)	1,335.8 (1,886.2)	2,314.8 (2,778.5)	<0.001
Cost ($) by department-other, mean (SD)	32,743 (43,414)	25,753 (33,799)	39,733 (50,297)	<0.001

After controlling for key covariates using a GLM, patients with anastomotic leaks had 0.8 times (*P* < 0.001) higher total costs (of index hospitalization and re-admission) than patients without leaks (Table 6).

**Table 6:** Generalized linear model for total cost of hospitalization plus re-admission

Variable	Category	Estimate-exp (B)	Confidence interval	*P* value
Intercept		40,733.373	(39,708.56, 41,784.63)	<0.001
Anastomotic leak (ref = no leak)		1.802	(1.78, 1.83)	<0.001
Age^a^		1.000	(1.00, 1.00)	0.222
Gender (ref = male)		0.969	(0.96, 0.98)	<0.001
Race (ref = white)	Black	1.079	(1.07, 1.09)	<0.001
	Hispanic	1.189	(1.17, 1.21)	<0.001
	Other	0.974	(0.97, 0.98)	<0.001
Payer (ref = Medicare)	Medicaid	1.065	(1.05, 1.08)	<0.001
	Commercial	0.984	(0.98, 0.99)	<0.001
	Other	0.982	(0.97, 1)	0.015
Medical stabilization (ref = no)	Yes	1.316	(1.31, 1.33)	<0.001
Admission type (ref = elective)	Urgent	1.039	(1.03, 1.05)	<0.001
	Emergency	1.05	(1.04, 1.06)	<0.001
	Other	1.041	(0.99, 1.1)	0.145
Type of index surgery (ref = left/sigmoid)	Right colon	0.273	(0.27, 0.28)	<0.001
	Colorectal	0.33	(0.33, 0.33)	<0.001
Severity of illness (ref = extreme)	Minor	0.483	(0.48, 0.49)	<0.001
	Moderate	0.273	(0.27, 0.28)	<0.001
	Major	0.33	(0.33, 0.33)	<0.001
Number of beds (ref ≥500)	<500 beds	1.062	(1.05, 1.07)	<0.001
Geographic location of provider (ref = midwest)	Northeast	1.129	(1.12, 1.14)	<0.001
	South	1.024	(1.01, 1.03)	<0.001
	West	1.165	(1.15, 1.18)	<0.001
Location of the hospital (ref = urban)	Rural	1.061	(1.05, 1.07)	<0.001
Teaching hospital (ref = no)	Teaching	1.083	(1.08, 1.09)	<0.001
Charlson Comorbidity Index^a^		1.005	(1, 1.01)	<0.001

^a^Age and Charlson Comorbidity Index are continuous variables with no reference groups

## Discussion

Anastomotic leaks remain a source of clinically significant postoperative complications and are a risk factor for increased morbidity and mortality. Our study sought to evaluate whether anastomotic leaks influence economic and clinical outcomes at a population level.

The overall incidence rate of anastomotic leaks in a 30-day postoperative period was 6.18 %; similar annual rates were observed for 2008, 2009, and 2010. Patients undergoing rectal surgery had a slightly elevated risk of leaks than those undergoing right or left colon surgery. This is in agreement with previous studies showing an increased incidence of leaks in the rectal area.^15^–^18^ A prospective study examining 1,834 patients reported higher leak rates in patients undergoing rectal/rectosigmoid surgeries (6.7 %) as compared to colonic anastomoses (2.6 %).^3^ Regardless of location, these leaks were often identified either in the late postoperative period or after discharge. This might be attributed, in part, to hospitals adapting early discharge protocols.^19^, ^20^ Our results also showed differences in LOS and subsequent discharge for the two cohorts, suggesting that the actual burden of leaks is larger than the numbers captured in our analysis. However, our study did not show statistically significant differences in mortality between the two cohorts after PSM was applied. We believe that this is due to nonleak-related mortality among high-risk patients selected through PSM.

Diagnosing an anastomotic leak in an outpatient setting may result in delayed intervention and may possibly adversely affect patient outcomes.^9^, ^21^ In our study, 27 % of patients with anastomotic leaks had infections as compared with 9 % of patients without leaks (Fig. 2). We used two definitions for postoperative infection—one based on ICD-9 codes 998.5X/998.6X and the other based on nonprophylactic antibiotic usage as a proxy for postoperative infections, defined as postoperative exposure to predefined antimicrobial drugs 2 days after surgery and with treatment duration ≥7 days. Using prophylactic antibiotics as a proxy for postoperative infection is previously documented—Yokoe et al. state that for many clinicians, administering antibiotics to patients who have recently undergone surgery indicates a suspicion of surgical site infections and may indeed accurately reflect the presence of infection when microbiologic confirmation is not obtained.^12^ In our study, patients with leaks showed 0.8–1.9 times higher postoperative infections than patients without leaks; this is consistent with previous studies.

In terms of economic impact, total inpatient costs were considerably greater for patients with leaks as compared to patients without leaks. Based on our results, patients with leaks tend to spend almost a week more in a hospital, with average incremental costs of $24,129 incurred for hospitalization alone. Increased LOS in patients with leaks is an expected result of the increased clinical sequelae. However, earlier studies have used varying definitions for anastomotic leaks. Our approach for defining leaks was unique in that our focus was on all clinical anastomotic leaks as opposed to only clinically *significant* anastomotic leaks (*significance* as defined by the authors) or inclusion of occult leaks, defined in earlier literature. While previous studies have defined leaks of clinical significance, we believe any leak is significant by virtue of being clinically apparent and requiring intervention. Nonclinical leaks are occult and unseen, unless a routine (and often unwarranted) scan is done, and are unlikely to seriously influence morbidity and mortality. Bruce et al. have explored the option of defining subclinical leaks;^22^ however, criteria for defining these remain unclear. We eliminated subclinical anastomotic leaks, as lack of clinical signs and symptoms translates into an occult leak.

In terms of patients requiring subsequent interventions, we identified abdominal re-interventions within 2–30 days as opposed to within 3–45 days postsurgically. We defined re-intervention—as in the ICD-9 procedure codes and Premier Perspective Charge Master codes—by re-operation, percutaneous drainage, stents, and radiology with drainage; radiology was not entered as a stand-alone code, but coupled with drainage. These re-interventions result in increased LOS or re-admission. This slightly modified identification procedure helped us analyze LOS and re-admissions in a more definitive manner. Results for increased LOS were consistent with previously reported rates.

A study evaluating 56 patients undergoing colorectal surgery found that patients showing symptomatic or clinical evidence of anastomotic leaks had significantly longer durations of hospital stay when compared with those without leaks (14 ± 1.41 vs 5.43 ± 0.89 days for patients with leaks and without leaks, respectively).^6^ Another recent study by Hashemi et al. sought to assess the economic impact of anastomotic leaks after colectomy procedures.^23^ As in our study, this study used information from the Premier Perspective™ database and examined data from 2005 to 2009 of 46,788 patients with anastomotic leaks. Results for mean hospital LOS were in agreement with our findings; patients with leaks spent approximately 7 days more in a hospital than those without leaks (14.9 vs 8.4 days). This study also reported an increase in leak rates observed between 2005 and 2009 from 16.2 to 22.1 %. Further, mean costs per discharge and overall inpatient costs were greater for patients with anastomotic leaks.

While there were similarities in findings between the previously mentioned study and our study, some core differences can be highlighted. The first is a variation in the methodologies used to identify the leaks themselves. The Hashemi study did not specify what surgical procedures were selected among ICD-9 codes, while our study specifically defined re-intervention procedures such as re-operation, re-anastomosis, stent, colostomy, drainage, and abscess within a 30-day postoperative window, in addition to identifying procedures by ICD-9 codes, CPT codes, and Perspective Standard Charge Master codes. Another key differentiator for our study was the use of both univariate and multivariate analyses, and the GLM and PSM methods used for statistical analyses. The use of multiple statistical methods and examination of matched and unmatched data for costs associated with LOS provides further evidence of the considerable economic burden associated with anastomotic leaks, in addition to the attendant increase in morbidity.

In this study, we used both PSM and GLM methods to assess the effect of leaks on total costs by controlling for observed confounders, and the results are consistent with each other. These two methods differed in the assumptions and analysis power (GLM assumed a linear structure of data and PSM excluded control patients who did not meet the matching criteria). Lastly, our study took into account re-admission due to anastomotic leaks. While the previously mentioned studies by Hashemi et al. and Fouda et al. have shown the significant impact of anastomotic leaks on total costs, they did not examine the extent to which patients with leaks were readmitted and the impact of re-admissions on costs. Re-admission places significant increases in cost on an already burdened healthcare system. Dor et al. estimate that the average unadjusted cost for colectomy alone stands at $21,257; this number would increase considerably when postsurgical complications such as leaks are added,^24^ as observed in our study. Our results show that patients with anastomotic leaks had a 1.3-fold greater chance of re-admission within a 30-day period.

With our findings and those of others as evidence, it can be safely assumed that prevention of leaks can save cost and clinical burden. Potential cost reductions from preventing anastomotic leaks could lead to a more judicious use of hospital resources, including an increased focus on training.^25^ The total burden of leaks in terms of LOS per 1,000 patients was 16,800 and 26,300 days for patients with no leaks and with leaks, respectively (Fig. 3a). Furthermore, the average total burden of costs per 1,000 patients was $44.3 million in patients with no leaks as compared to $72.9 million in patients with leaks (Fig. 3b). Based on our results, it seems evident that there is a significant impact of anastomotic leaks on re-admission, LOS, and total costs.

While the present study does point toward the advantages of preventing anastomotic leaks, there are a few potential limitations. Our study had a retrospective study design and did not cover the management of anastomotic leaks. Furthermore, the Premier Perspective™ database is only representative of inpatient data from the USA. Thus, the results from this study cannot be extrapolated as a representation of the economic burden of anastomotic leaks in other countries. Additionally, the classification of standardized outcome measures may have prevented analysis from any detailed procedure-specific outcomes. Furthermore, more patients with anastomotic leaks were discharged from the hospital to a nursing facility other than a home. Due to the lack of outpatient data in these cases, mortality may be underestimated for patients with leaks, and this could be a cause of the small difference observed in the in-hospital mortality between patients with and without leaks. Additionally, matched control patients (without anastomotic leaks) had a relatively high APR-DRG disease severity level, a strong predictor of mortality; therefore, this could be a factor in the relatively high observed mortality for matched controls. Lastly, another potential limitation of this study is that we did not consider open versus laparoscopic procedure as a study variable.

## Conclusion

In conclusion, anastomotic leaks following colorectal surgery increase the total clinical and economic burden by a factor of 0.6–1.9 in terms of additional 30-day re-admission, postoperative infection, LOS, and hospital costs. The results of this study underscore the potential advantages of cost reduction for patients and hospitals by preventing anastomotic leaks after colorectal surgery. The prevention of postoperative anastomotic leaks must remain a priority for healthcare providers; this will ease a significant clinical and economic burden.
